# A systematic review on the effectiveness and impact of clinical decision support systems for breathlessness

**DOI:** 10.1038/s41533-022-00291-x

**Published:** 2022-08-20

**Authors:** Anthony P. Sunjaya, Sameera Ansari, Christine R. Jenkins

**Affiliations:** 1 Respiratory Division, The George Institute for Global Health, Sydney, NSW Australia; 2 Faculty of Medicine and Health, UNSW Sydney, Sydney, NSW Australia; 3 School of Population Health, Faculty of Medicine and Health, UNSW Sydney, Sydney, NSW Australia; 4 Faculty of Health Sciences and Medicine, Bond University, Gold Coast, QLD Australia

**Keywords:** Health services, Health care economics, Respiratory signs and symptoms

## Abstract

Breathlessness is a common presenting symptom in practice. This systematic review aimed to evaluate the impact of CDSS on breathlessness and associated diseases in real-world clinical settings. Studies published between 1 January 2000 to 10 September 2021 were systematically obtained from 14 electronic research databases including CENTRAL, Embase, Pubmed, and clinical trial registries. Main outcomes of interest were patient health outcomes, provider use, diagnostic concordance, economic evaluation, and unintended consequences. The review protocol was prospectively registered in PROSPERO (CRD42020163141). A total of 4294 records were screened and 37 studies included of which 30 were RCTs. Twenty studies were in primary care, 13 in hospital outpatient/emergency department (ED), and the remainder mixed. Study duration ranged from 2 weeks to 5 years. Most were adults (58%). Five CDSS were focused on assessment, one on assessment and management, and the rest on disease-specific management. Most studies were disease-specific, predominantly focused on asthma (17 studies), COPD (2 studies), or asthma and COPD (3 studies). CDSS for COPD, heart failure, and asthma in adults reported clinical benefits such as reduced exacerbations, improved quality of life, improved patient-reported outcomes or reduced mortality. Studies identified low usage as the main barrier to effectiveness. Clinicians identified dissonance between CDSS recommendations and real-world practice as a major barrier. This review identified potential benefits of CDSS implementation in primary care and outpatient services for adults with heart failure, COPD, and asthma in improving diagnosis, compliance with guideline recommendations, promotion of non-pharmacological interventions, and improved clinical outcomes including mortality.

## Introduction

About 10% of Australian adults are reported to have breathlessness^1^. Greater prevalence has been reported in specific groups such as the elderly, with ~27–47% being breathless depending on the population^2^.

In light of the high prevalence of breathlessness and its associated under-reporting, primary healthcare (PHC) plays an essential role in its recognition, diagnosis, and management^3^ However, diagnosing the cause of breathlessness can be difficult due to the myriad possible etiologies, ranging from pulmonary to cardiac, metabolic diseases and deconditioning. In the Australian primary care setting^4^, it was reported that <30% of patients with breathlessness had a referral diagnosis fully concordant with the final diagnosis. This finding was supported by a Danish study where in those referred with suspected heart failure, the diagnosis was confirmed in 31%, and altogether, only 39% of the patients referred for breathlessness had a final diagnosis in concordance with the referral diagnosis^5^. Clinical algorithms can help with this and a previous review^6^ reported that through the use of simple tests it is possible to elucidate the diagnosis of ~50% that presented with chronic breathlessness.

In many patients, breathlessness can be attributed to either medical or lifestyle problems which are treatable and preventable^7^. However, when these patients are misdiagnosed, inappropriate diagnostics and ineffective treatment plans can result in overuse of medications, potentially serious side effects, cost to patients and the community, and wasted opportunity to prevent morbidity and address lifestyle issues.

Time constraints in primary care contribute to the low concordance for final diagnosis. One-third of primary care physicians are dissatisfied with the time available per patient and suggest this compromises the care they provide^8^. Under time pressure, primary care physicians were reported to ask significantly fewer questions and conduct less thorough clinical examinations^9^.

Furthermore, studies indicate that despite a growing abundance of disease-specific guidelines, they are frequently not applied, resulting in unnecessary diagnostic tests and inadequate or potentially harmful treatments^10^. An estimated 30–40% of patients receive non-evidence-based treatments of which 20–25% are not needed or are potentially harmful^11^. Perez et al. in an American study of COPD guidelines adherence by 154 primary care physicians reported only 5% adhered to referral guidelines, ~20% to pulmonary function test guidelines for smokers, and ~50% to treatment guidelines^12^. In a recent study among asthma patients in Canada, only 4% of primary care physicians consistently reported providing a written Asthma Action Plan (AAP)^13^ with a previous study reporting only 2% of asthma patients actually received one^14^.

Clinical decision support systems (CDSS) are defined as any electronic system designed to directly aid clinical decision-making that can help generate patient-specific assessments or recommendations which are presented to clinicians for consideration^15^. Previous studies on other health conditions such as gastrointestinal disease and critical care have suggested that CDSS can result in significantly safer prescribing decisions and closer adherence to recommended guidelines compared to their peers using paper resources^16^. The World Health Organization (WHO) has identified the development of health information systems and digital technologies including CDSS as one of the priorities to strengthen PHC^17^. However, research on effective and applicable approaches to utilize CDSS to strengthen PHC remains scarce^18^.

To date, we have been unable to find any systematic reviews assessing the use of CDSS for breathlessness patients in primary care and outpatient services where they may provide the most impact. Hence, this systematic review aimed to investigate the evidence for CDSS in assessing and managing breathlessness and its applicability to primary care. We also sought to identify features that are associated with beneficial outcomes as well as unintended consequences in real-world practice.

## Methods

The protocol was prospectively registered and published in PROSPERO (CRD42020163141). The Preferred Reporting Items for Systematic Reviews and Meta-Analyses (PRISMA) were used in the reporting of this study.

### Selection criteria and search strategy

A comprehensive search of databases was conducted in consultation with an independent research librarian. We searched 14 research databases including CENTRAL, Embase, Pubmed, clinical trial registries, and Epistemonikos to obtain relevant systematic reviews and undertook hand searching from reference lists to identify potentially eligible primary studies. Studies were limited to those published between 1 January 2000 to 10 September 2021 and in the English language. The list of databases and keywords utilized are available in Appendix 1 and Supplementary Table 1.

Studies were included if they compared both digital and non-digital CDSS with no intervention or usual care, in the primary care setting or through first-contact outpatient services. These could include clinics delivering care to patients with breathlessness in general and most likely associated diseases—asthma, COPD, heart failure, obesity/deconditioning, and psychogenic breathlessness. A CDSS was defined as any system designed to aid directly in clinical decision-making, in which characteristics of individual patients are used to generate patient-specific assessments or recommendations that are then presented to clinicians for consideration^15^. We excluded studies where patients were the sole users of the CDSS, studies of CDSS solely used by specialists, and studies where the CDSS was used solely for training, without real-world healthcare delivery.

### Data collection

Two review authors (AS and SA) independently performed the selection of studies. All titles and abstracts retrieved were downloaded to a reference manager (Endnote X9 for Windows, Thomson Reuters, Philadelphia, USA) where duplicates were removed. The screening was then done independently by two reviewers (AS and SA) using the open-source web tool, Rayyan QRCI^19^. Similarly, full-text articles of all studies that were screened and included were downloaded and then screened against the inclusion and exclusion criteria through Rayyan QRCI. Reasons were documented for excluding studies.

Risk of bias was assessed using the Cochrane Risk of Bias v2 (RoB v2) tool for randomized clinical trials (RCT), Risk Of Bias In Non-randomized Studies—of Interventions (ROBINS-I) tool for observational studies, and Quality Assessment of Diagnostic Accuracy Studies (QUADAS-2) for diagnostic studies in accordance with the Cochrane Handbook for Systematic Review of Interventions^20^ and guidelines for review from the Cochrane Effective Practice and Organisation of Care (EPOC) group^21^. Due to the nature of the intervention being integrated with care, the non-blinding of provider and participants did not result in an automatic increased risk of bias. Data extraction was conducted using a standard electronic form using the Systematic Review Data Repository-Plus (SRDR+) tool. All disagreements were resolved through discussion, or if required, the outcome was decided by a third reviewer. Results of the extraction were narratively reported in accordance with the Cochrane Handbook for Systematic Review of Interventions^20^.

### Outcomes

The main outcomes of interest were patients’ or clients’ health outcomes, assessed through validated measures; providers’ use and adherence to recommendations, guidelines, or protocols (eg appropriate referrals, management); time and test efficiency and diagnostic accuracy or concordance between the CDSS diagnosis and final diagnosis.

Other outcomes of interest include physicians’ acceptability and satisfaction with the intervention; patients’ or clients’ acceptance of and satisfaction with the intervention; economic evaluations; reports of impact on resource use; quality of data and unintended consequences.

## Results

**Fig. 1:**
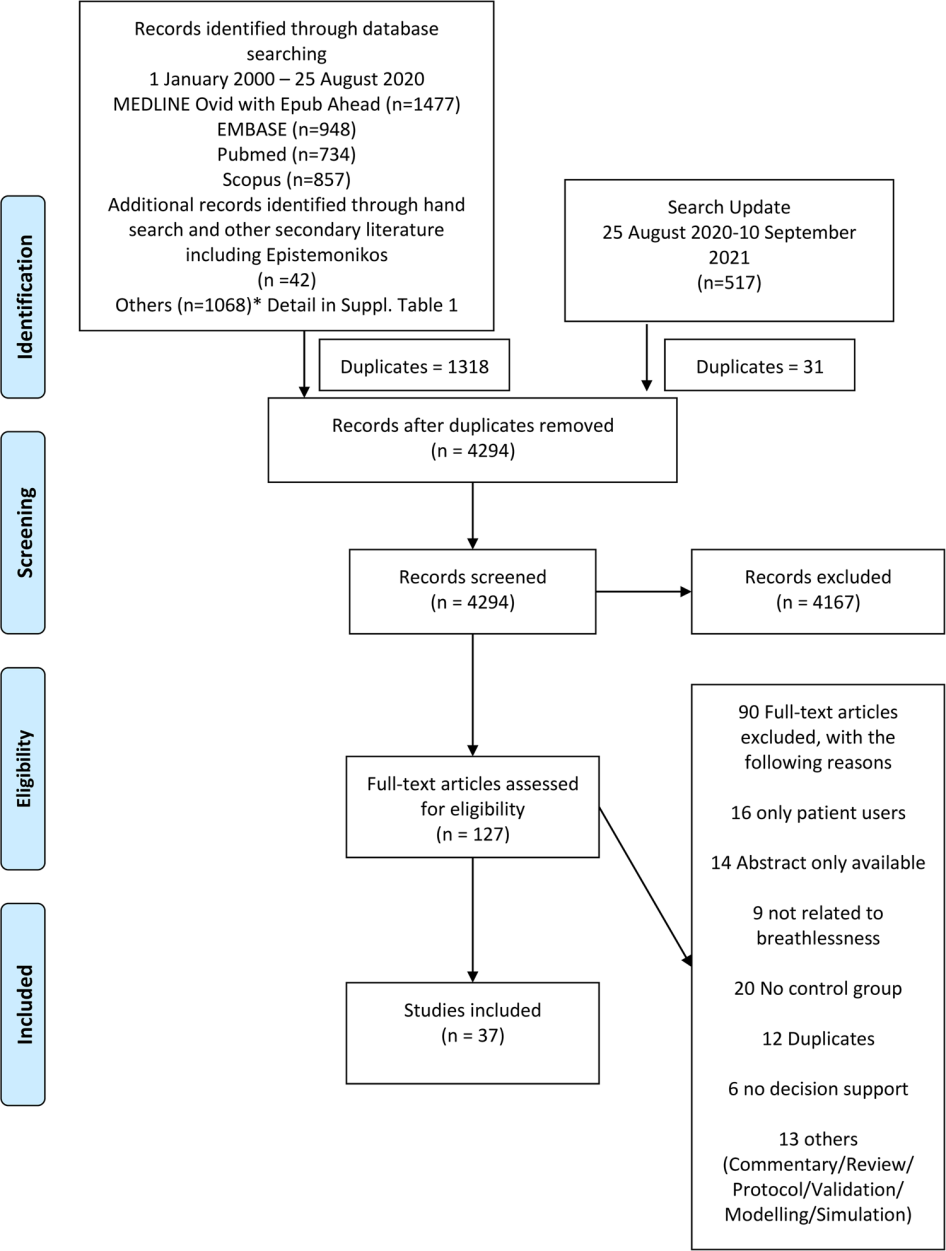
PRISMA flow diagram^59^.

A total of 4294 records were screened, 127 underwent full-text review and 37 studies were included (Fig. 1). Many studies were excluded as they report only retrospective validation^22^ or was purely for patient use and did not inform clinicians^23^. Majority of the studies (*n* = 30) included were RCTs. Study duration ranged from 2 weeks^24^ to 5 years^25^. About 55% of the studies (*n* = 20) were conducted in the United States followed by the Netherlands (*n* = 6)^26–31^ and three each in Canada^32–34^, South Korea^35–37^, and United Kingdom^38–40^. All were in high-income countries and mostly in urban settings (Supplementary Table 2).

Most studies were conducted only in primary care or had a primary care arm (*n* = 20), followed by 13 in the ED and four in a mixed setting. CDSS interventions described in the studies varied from non-electronic support to alerts, expert systems, and artificial intelligence-based CDSS. About half (*n* = 17) referred to either hospital, national or international guidelines.

Only one study explicitly addressed breathlessness. The majority were for asthma (*n* = 19) or heart failure (*n* = 9). Most focused on a single disease with seven addressing more than one disease.

### Outcomes from CDSS for multimorbidity

Majority of the studies (6/8) were cluster RCTs and conducted in primary care. Some CDSS focused on a combination of diseases such as asthma and angina, others on symptoms such as breathlessness and chest pain, and on diagnostic test support such as spirometry. (Supplementary Tables 2 and 3).

Studies reported mixed results. In studies with no significant difference in the CDSS arm compared to control arm, a low level of software use (median active interactions with the CDSS = 0)^40^ and large variations between GPs’ prescribing behavior which underpowered studies^28^ was reported. Process outcomes (guideline compliance) were reported to significantly improve in a cardiovascular CDSS study^41^, but an earlier study reported otherwise^42^.

In the emergency department (ED), a multi-center RCT by Kline et al.^43,44^ reported no significant difference in median length of stay in the ED, but median length of stay in the hospital was significantly lower in the intervention group (7.7 h [IQR 4.0–27.3] vs 8.9 h [IQR 4.8–29.6], *p* = 0.046).

### Outcomes from asthma-specific CDSS

Eighteen studies found were focused on asthma. There was an equal mix of those conducted in children and adults.

In children, CDSS use for diagnosis was found to improve the proportion diagnosed with asthma^45^ but reported mixed impact on exacerbations^46,47^, symptom days^48^ and influenza vaccination rates^49^ post-implementation. However, a pilot study^50^ reported improvement in patient-reported outcomes such as missing days from work and quality of life. A few studies focused on management, especially measuring adherence to guideline recommendations. Some studies showed improved adherence to guidelines on spirometry^51^, AAPs^51^, peak-flow measurements^29^, and oxygen saturation measurement^24^.

In adults, a diagnostic study^37^ reported a CDSS sensitivity of 85.2% and specificity of only 25% in ascertaining a diagnosis of asthma. As in children, several studies have reported improved guideline adherence in the CDSS arm. Studies reported improvement in peak-flow measurements^29^, inhaled corticosteroids (ICS) prescription^32,36,52^, and use of AAPs^52^. They also reported improved symptoms, Asthma Quality of Life Questionnaire (AQLQ) score, forced expiratory volume one second (FEV-1) value, airway hyperresponsiveness^52^, and reduced exacerbations^39^. The benefits on hospitalization were mixed ^33,39^.

### Outcomes from COPD-specific CDSS

Two studies^26,53^ focused on patients with COPD. Both reported improvements in patient-reported outcomes upon CDSS implementation.

### Outcomes from heart failure-specific CDSS

Eight CDSS focused on heart failure patients, for various uses ranging from diagnosis to management as well as being linked to telemonitoring systems. A diagnostic study using an artificial intelligence-based CDSS^35^ found a significantly higher accuracy of the CDSS compared to usual care (97.9% vs 76.3%). Studies also showed benefits on improving cardiac rehabilitation^31^ and management guideline compliance^54^.

One RCT^54^ among patients with heart failure reported significantly lower mortality in the CDSS compared to control group (2% vs 14%), although no significant differences in hospitalization and ED visits were found between groups. Another RCT^55^ reported a paradoxical increase in all-cause hospitalizations which the investigators attributed to similar levels of guideline adherence between intervention and control groups (33% vs 30%, *p* = 0.4).

Two studies^30,38^ evaluated integrating CDSS with telemonitoring. Both reported no significant difference in outcomes, but one did show a significantly lower number of heart failure-related outpatient clinic visits (median 2 vs 4, *p* = 0.02)^30^.

### Health economic outcomes

The cost of managing breathlessness can arise from both direct costs (healthcare use, medication costs) and indirect costs (productivity loss)(Supplementary Table 4). The majority of studies (seven) reporting this outcome focused on asthma, with two on heart disease and one on breathlessness in the ED.

For asthma, studies reported lower number of missed days at work and school in the CDSS group than control group^48,50^, with mixed results on the impact on the number of health visits^29,39^. One cost-benefit analysis^48^ reported that by year 1, the savings (USD337/child) from the reduction in ED visits from a CDSS intervention were sufficient to provide a cost-benefit. Montecarlo simulation suggests a 97% chance of this cost saving. Other studies reported mixed results, some reported no difference^46^, while others a higher cost^24,56^ in the intervention group with wide variations in cost.

Two studies on patients with heart disease reported a cost-effectiveness analysis. One^30^ reported that the addition of telemonitoring did not lead to greater cost-effectiveness. Another^42^ reported that while total health charges were lower for the intervention compared to control group, this was not statistically significant due to the wide variation in outpatient and inpatient costs reported in the study.

One multimorbid CDSS study conducted in the ED^43,44^ reported significantly lower median costs for medical care within 30 days in the intervention compared to the control group with small lifetime gains in Quality Adjusted Life Years.

### Effect on physicians

Various studies assessed the impact on physicians differently (Supplementary Table 5). Studies reported that CDSS use improved knowledge^33,36^ and were found to be useful for daily practice^33^. Low rates of CDSS use were however reported in many studies^41,55^. High inter-doctor variation in prescribing behavior was found^28^ and providers were reported to be more compliant to reminders barring prescription of certain drugs rather than those that recommend certain medication.

Qualitative studies^39^ report that management recommendations and reminders were popular with the users and were deemed to provide relevant clinical advice. Physicians were reported to differentially use CDSS and were more likely to use it in out-of-control patients^32^. Physicians’ attitude towards guidelines underlying clinical recommendations were mixed. They ranged from viewing guidelines as providing helpful information but not helpful in making decisions for individual patients^42,56^ to CDSS recommendations not meeting patient needs^25,41,55^. Studies suggest that GPs have differing needs and that GPs handling more complex conditions may be more amenable to using CDSS^40,41^.

Despite this, studies also show that CDSS can save time^41,46^ and that providers had the impression that CDSS allowed them to accomplish more work than would otherwise be possible^41^. Although visits tend to last longer in the CDSS group, a CDSS can still be used in consultations lasting <10 min^39^.

### Effect on patients

Studies evaluated patient satisfaction through various measures. In patients with heart disease, Tierney et al.^42^ found no significant difference in quality of life, medication compliance, and satisfaction with care between the intervention and control groups. Similarly, Breathett et al.’s study^57^ in heart failure patients found that while median patient satisfaction was higher in the intervention group, this was not statistically significant (*p* = 0.08). Even so, the study reported that patients particularly felt providers offered better explanations of their care in the intervention than control arm (83.7%, vs 55.8%, *p* = 0.01). They reported that patients in the intervention group perceived significantly better descriptions of medication side effects than the control group (61.1% vs 26.7%, *p* = 0.01).

Subramanian et al.^55^ study among heart failure patients also reported similar results. At six months, patients in the intervention group were more satisfied with their physicians (*p* = 0.02) and primary care visit (*p* = 0.02). However, at 12 months, only satisfaction with the most recent primary care visit remained statistically significant (*p* = 0.01).

Slok et al.^26^ in a study on COPD patients utilizing the Patient Assessment of Chronic Illness Care (PACIC; a measurement of perceived quality of care) score reported a 0.32 point (95% CI 0.14 to 0.50) improvement in the intervention group (scores range from 1–5).

In contrast, asthma patients in three studies assessing patient satisfaction reported no significant difference in activation score^50^, partnership problems^58^, or satisfaction in general^56^. The use of a patient-facing CDSS kiosk was reported to not improve partnership with providers^58^. The authors report that providers’ inattention to parents’ concerns communicated via the kiosk may explain a trend toward worsening partnerships noted in the adjusted results. Improvements in information sharing scores only occurred in the subset of patients whose kiosk output was acted upon by providers. The use of a CDSS for breathlessness in the ED^43,44^ was also reported to not improve patient satisfaction survey score (*p* = 0.148).

### Unintended consequences of CDSS use on clinical practice

None of the studies reported serious adverse events resulting in death or breach of confidentiality in both intervention and control groups^46^. However, there were differential impacts of CDSS implementation on patients depending on their location^51^, age^29,33^, and regularity in visiting a health facility^33^ (Supplementary Table 6) This relates to findings from other studies^29,31^ which discussed how system-wide factors are also possible limitations to CDSS effectiveness. Furthermore, studies have also reported an increase in unscheduled visits^52^, and both higher dosing^52^ and underdosing^54^ of patients. For those with a patient-facing component, lowering engagement was also reported ^34^.

### Risk of bias

The majority of RCTs were classified as low risk of bias (21/30 studies); eight had some concerns and one had a high risk of bias. For observational studies, three were assessed to have a serious risk of bias, one moderate risk, and one low risk. For the two diagnostic studies, one was assessed to be high risk and another low risk. Further details are in Appendix 2.

## Discussion

This review found conflicting results with regard to the effectiveness of multimorbid CDSS in primary care and outpatient services. However, in disease-focused CDSS, improved guideline compliance was found. These CDSS for COPD, heart failure, and asthma in adults reported clinical benefits such as reduced exacerbations, improved quality of life, improved patient-reported outcomes or reduced mortality. CDSS for asthma in children reported mixed results. Only one CDSS was explicitly for breathlessness as a symptom which was implemented in the ED and generated positive results.

For CDSS focused on asthma in children, studies reported increase in diagnosis compared to usual care^45^, guideline-compliant prescription^51^, and adherence to diagnostic recommendations^24,29,51^. However, while CDSS were reported to reduce odds of exacerbations in some of the studies^47^, no significant differences in healthcare use (ED visits, hospitalization, etc.) were reported.

In adults, studies suggest that CDSS for asthma not only improve guideline compliance^29^ but also reduce exacerbations^39^, GP visits^39^, and ED visits^33^ with mixed results for hospitalization. Whereas for COPD, CDSS were found to improve SGRQ^26^ but not CAT scores. Furthermore, inpatient, outpatient, and total exacerbations were found to be lower post-intervention^53^.

CDSS for heart failure was shown to be able to provide comparable diagnostic accuracy to heart failure specialists^35^ and improve access to cardiac rehabilitation^31^. We note that the diagnostic study was only conducted in a single center and would require further validation in other settings. A stark sevenfold reduction in mortality in the CDSS arm compared to control arm was also reported in one of the RCTs^54^, stemming from greater prescription of beta-blockers. However, most studies reported no significant differences on hospitalization rates^25,54,57^. Addition of telemonitoring in the two studies that reported them did not suggest a benefit for mortality or quality of life^30,38^, but did significantly lower outpatient visits^30^ in one of the studies which might justify the additional costs of this added peripheral.

There were few studies reporting cost-effectiveness, results ranged from potential savings to higher medical costs. One study^48^ was able to show that even a reduction in one component of healthcare use, i.e., ED visits, was able to recoup the cost of CDSS intervention. Furthermore, it is important to consider a holistic view with regards to cost benefit; while costs may increase in the short term due to setting up and increased compliance with guidelines in the number of visits, diagnostics, and management, the improved clinical outcomes and mortality benefit would lower costs in the long run beyond the trial period.

Providers were found to respond differently in the various studies. While in general they were satisfied and had improved knowledge post-CDSS intervention, there was a discordance between this and compliance with recommendations. Low compliance of <50% across many studies was found, implying that the CDSS evaluated were not fit for purpose, trusted by clinicians or may have been difficult to use. Other possible explanations for low use include selective CDSS use only in difficult cases^32^ and where CDSS promote guideline-directed management, this was generic and not specific enough for patient use.

Providers had mixed attitudes towards guidelines, which would affect uptake and efficacy of CDSS, beyond factors from the CDSS itself. Increasing trust in the evidence underlying CDSS recommendations may be a mechanism to promote greater uptake. Recommendation presentation was also important. Differential adherence to CDSS recommendations was reported with those recommending ceasing prescription of medication due to lack of indication being followed more than those recommending starting new medication^28^.

Hence, even a CDSS with a true positive effect will be biased towards null as both the intervention and control arms may be minimally different with regards to intervention exposure. Even so, this finding in mostly clinical trial settings is concerning, as real-life practice would likely have even lower levels of use. Hence, there is a need to design CDSS that are fit for purpose, designed through user engagement and pilot testing with end users (providers and patients) to improve uptake.

Health system-wide factors were found to impact CDSS success. Time constraint is one of the main barriers to CDSS use in clinical practice, despite the intention that CDSS achieve time efficiency. Some studies showed potential time savings with CDSS use and while one reported longer visits, it was possible to employ a CDSS in consultations lasting less than 10 min. The use of CDSS by allied health professionals as well as doctors has been found to improve clinical outcomes, thus not necessarily extending consultation time.

Unfamiliarity with CDSS resulted in a higher proportion of 30–60-minute visits in the CDSS arm, potentially magnified by the use of older technology in that study^24^. A longer consult time might also relate to better explanations of symptoms and management of patients in the CDSS group, prompted by the CDSS^55,57^. Improvement in patient satisfaction was however mixed between studies and one study with a patient-facing component of the CDSS found improvement in satisfaction only when patients’ inputs to the CDSS were acted upon.

From a system perspective, CDSS use was reported to improve providers’ recording practice^29,55^, which is important in relation to future use of the labeled data for machine learning, algorithm development, etc. Furthermore, one study reported the sustainability of use beyond the period during which financial incentives were offered to providers for CDSS use. Although CDSS may have limitations, many of these can be anticipated and addressed by providing context-specific knowledge to improve usability and outcomes.

In general, the implementation of CDSS in practice was found to be safe. It is important that the underlying algorithms address differences but minimize rather than widen inequity between clinical and demographic groups. It must also be noted that guideline compliance as a consequence of CDSS may increase medication use and achieve greater benefits but cause more side effects from treatment thus not always translating to better clinical outcomes. However, CDSS does have the potential to encourage the prescription of non-pharmacologic management such as lifestyle changes which can provide a more holistic clinical impact with fewer side effects. Although of note the greater resource needs such as nutritionists, physiotherapists, and psychologists to deliver these non-pharmacologic interventions which might not be available in a health system. Leveraging evidence-based mobile apps for self-management linked with CDSS maybe one avenue to bridge this resource gap.

A strength of this review is the comprehensive database search conducted and the inclusion of high-quality studies which were assessed to be mostly at low risk of bias. However, despite this comprehensive search a limited number of studies were found that address the multimorbid nature of breathlessness. Furthermore, the wide variation in CDSS purpose and function across studies also precluded quantitative assessments of effect sizes to be made. However, the review summarizes not only the impact of CDSS on clinical outcomes but also examines health economic impact, its effect on physicians and patients as well as unintended consequences of their implementation in practice.

Future studies should focus not only on process outcomes but on hard clinical endpoints including mortality as well as outcomes of interest to the health system such as health service utilization, medication, and hospitalization costs. Furthermore, future studies should assess how CDSS may integrate with evolving modes of healthcare delivery such as telehealth and virtual care services that will likely form a substantial portion of future health systems.

This review found no breathlessness-focused CDSS but identified evidence of benefits of CDSS implementation in primary care and outpatient services. This included improving diagnosis, greater compliance with guideline recommendations, promotion of non-pharmacologic interventions, and improved clinical outcomes including mortality for adults with heart failure, COPD, and asthma. However, clinician inertia and logistical barriers to implementing CDSS recommendations remain critical barrier to success. This should be balanced with the suitability of background algorithms and management recommendations which most commonly stem from guidelines that may not reflect the clinical variation and facilitate the precision of care that many clinicians seek to achieve. Studies have shown that CDSS are safe and cost-effective while their impact on patient satisfaction and healthcare use were mixed. Future studies need to focus on improving uptake in practice and developing of underlying algorithms that fit closer to clinical practice needs.

## Supplementary information

Manuscript Supplement

## Data Availability

The authors declare that all data supporting the findings of this study are available within the paper.
