# Supplementary material for: A systematic review on the effectiveness and impact of clinical decision support systems for breathlessness
Source: NPJ Prim Care Respir Med. 2022 Aug 20;32:29. doi: 10.1038/s41533-022-00291-x (PMC9392800; doi:10.1038/s41533-022-00291-x)
Supplement: Supplementary file 1 — Manuscript Supplement [file 41533_2022_291_MOESM1_ESM.docx]

**SUPPLEMENTARY INFORMATION**

**Effectiveness and impact of clinical decision support systems for breathlessness: A systematic review**

**Review team members:** Anthony Paulo Sunjaya (AS), Sameera Ansari (SA), Christine Jenkins (CJ)

**Affiliations:** The George Institute for Global Health, School of Population Health, UNSW Sydney and Faculty of Health Sciences and Medicine, Bond University

**Conflicts of interest:** None

**Appendix 1 - Search Strategy**

**Database serving as example**: Medline Ovid

| **Concept** | **Keywords, Synonyms** | **Related Terms** | **Subject Headings [MeSH]** |
| --- | --- | --- | --- |
| **Electronic Clinical Decision Support** | Expert system  Information systems  point of care system  reminder system  reminder system*  reminder system: and comput*  Medical Order Entry Systems  Decision support  Decision support ADJ3 system  Decision support ADJ5 system  Decision Support Techniques  Decision making, computer-assisted  decision support systems, clinical  Electronic Medical Record  Electronic Health Record  Tool  Aid  Comput* decision aid  Comput* decision making  clinical decision support aid*  System  Technique  Guide  clinical decision support guideline*  CDSS  CCDSS  CCDS  Clinical decision support system  clinical decision support system*  Comput* assisted adj2 therapy  Comput* assisted diagnosis  Diagnosis, Computer-Assisted  computer-assisted and drug therapy  computer-assisted and diagnosis  computer and diagnosis  computer-assisted and decision  computer and drug-therapy  computer assisted therap*  Therapy, Computer-Assisted  computer and therapy  computer and decision  comput* and decision support*  Computerised  Computerized  Computer-assisted  Computer-based  Computer-aided  Online  Web-based  Digital  Electronic  Electronic* | Software | exp Decision support system/  exp Clinical decision support system/  exp Reminder Systems/  exp Expert system/  exp Information system/  exp Medical Informatics/  exp Medical Information System/ |
| **Breathlessness** | Breathlessness*  Shortness of breath  Short of breath  Breath shortness  short* of breath  difficult* breathing  breathing difficult*  difficult repiration  Dyspne* or dyspneic  Dyspno* or dyspnoeic  Labored breathing  Dyspnea ADJ2 Effort  Dyspnoea ADJ2 Effort  Paroxysmal dyspnea  Dyspneic syndrome  Labored respiration  Laboured respiration  Pulmonary Dyspnea  Lung dyspnea  Lung dyspnoea | Breathing difficulties  Difficulty breathing  Tachypnea  Hyperventilation  Hypoventilation  Apnea  Bradypnea | exp Dyspnea/ |
| **Asthma** | Asthma  Asthma bronchial  Pulmonary asthma  Asthma pulmonale | Obstructive Airway Disease | exp Asthma/ |
| **COPD** | COPD  Chronic obstructive pulmonary disease  Chronic obstructive pulmonary disorder  chronic obstructive respiratory disease  COAD  Chronic Airflow Obstruction  chronic obstructive bronchopulmonary disease  Chronic obstructive lung disorder  Chronic Airway Obstruction  Pulmonary or Lung Emphysema  Chronic Bronchitis  lung chronic obstructive disease  lung disease, chronic obstructive  obstructive lung disease  obstructive lung disease, chronic  obstructive pulmonary disease  obstructive respiratory disease  obstructive respiratory tract disease | Obstructive Airway Disease | exp Chronic obstructive lung disease/  exp lung emphysema/  exp chronic bronchitis/ |
| **Heart Failure** | heart failure  backward failure, heart  cardiac backward failure  cardiac decompensation  cardiac failure  cardiac incompetence  cardiac insufficiency  cardiac stand still  cardial decompensation  cardial insufficiency  chronic heart failure  chronic heart insufficiency  decompensatio cordis  decompensation, heart  heart backward failure  heart decompensation  heart incompetence  heart insufficiency  insufficientia cardis  myocardial failure  myocardial insufficiency | Acute Heart Failure  heart outflow tract obstruction | exp Heart failure/  exp congestive heart failure/  exp diastolic dysfunction/  exp heart ventricle failure/  exp heart ventricle overload/  exp forward heart failure/  exp high output heart failure/  exp heart failure with preserved exp ejection fraction/  exp heart failure with reduced ejection fraction/ |
| **Obesity** | Obesity  Overweight | Overnutrition  Experimental obesity/  Lipedema/  Metabolic Syndrome X/  Metabolically benign obesity/ | exp Obesity/  exp Abdominal obesity/  exp Morbid obesity/  exp Obesity hypoventilation syndrome/  exp Adolescent obesity/  exp Childhood obesity/  exp Diabetic obesity/  exp Maternal obesity/ |
| **Deconditioning** | decondition*.mp.  cardiovascular deconditioning |  | exp cardiovascular effect/ |
| **Psychogenic Breathlessness** | Psychogenic Breathlessness.mp.  psychogenic dyspnea.mp.  hyperventilation syndrome.mp.  dysfunctional breathing.mp. | Psychosomatic disorder/  Hyperventilation/  Vocal Cord Disorder/ (Vocal Cord Dysfunction)  Mental Health Disorder  Depression  Anxiety | exp Hyperventilation syndrome/ |
| **Primary Care** | Primary care  First line care  General practice  Family practice  General physician  Primary care physician  Primary care doctor  Health clinic  Community health center/centre |  | exp Primary medical care/  exp primary health care/  exp general practice/  exp general practitioner/  exp family medicine/  exp health center/ |
| **Outpatient Services** | ambulant therapy  ambulatory treatment  ambulatory care  health care, outpatient  outpatient health care  outpatient service  outpatient therapy  outpatient care  emergency care.mp.  emergency healthcare | Emergency health service/ Triage | exp Outpatient care/  exp ambulatory care/  exp Emergency care/ |

Supplementary Table 1. Search result for the data sources

| **Data source** | **Number of records** |
| --- | --- |
| Embase | 948 |
| MEDLINE Ovid with Epub Ahead | 1477 |
| Pubmed | 734 |
| CENTRAL | 172 |
| Cochrane Library | 74 |
| Emcare | 485 |
| CINAHL | 16 |
| Web of Science | 272 |
| Health Technology Assessment Database | 1 |
| NHS Economic Evaluation Database | 31 |
| DARE (Database of Abstracts of Reviews of Effects) | 9 |
| ACP Journal Club | 8 |
| Clinical trials in trial registries (WHO ICTRP, Clinicaltrials.gov, ANZCTR) not already in MEDLINE | 0 |
| Epistemonikos and hand search of references | 42 |

*Note – mhealthevidence.org became defunct when we conducted the search and was not included. Clinical Trials (n=31) included as part of MEDLINE Ovid search.

Supplementary Table 2. Summary of Included Research Studies

| **Study** | **Country** | **Setting** | **Design** | **Participant** | **Duration (months)** | **RoB^#^** |
| --- | --- | --- | --- | --- | --- | --- |
| Ahmed et al 2016^1^ | Canada | Hospital | RCT | 18-69 years old, physician diagnosed asthma, prescribed at least 1 rescue medication, classified as having poor asthma control at the time of recruitment by their treating physician | 9 | Low |
| Ansari et al 2003^2^ | USA | Primary Care / General Practice Clinic | RCT | Heart failure patients, ejection fraction (EF) <45% and no contraindications to beta-blockers | 12 | Low |
| Bell et al 2010^3^ | USA | Primary Care / General Practice Clinic | RCT | Children (0-18 years) with asthma | 12 | Low |
| Breathett et al 2018^4^ | USA | Hospital | RCT | Heart failure patients | 1 | Low |
| Carroll et al 2012^5^ | USA | Primary Care / General Practice Clinic | RCT | Children with asthma aged 3 to 11 |  | Low |
| Cherney et al 2021^6^ | USA | Hospital | Cohort Study | Asthma patients | 12 | Moderate |
| Cho et al 2010^7^ | South Korea | Primary Care / General Practice Clinic, Hospital | Cohort Study | Asthma patients | 9 | Serious |
| Choi et al 2007^8^ | South Korea | Hospital | Cross Sectional | Outpatients with various respiratory symptoms such as dyspnea, cough or wheezing. |  | High |
| Choi et al 2020^9^ | South Korea | Hospital | Cross Sectional | Patients with dyspnea |  | Low |
| Dexheimer et al 2014^10^ | USA | Hospital | RCT | Children with asthma (2-18 years) | 5 | Low |
| Eccles et al 2002^11^ | United Kingdom | Primary Care / General Practice Clinic | RCT | Adult asthma and angina patients |  | Low |
| Esposti et al 2012^12^ | Italy | Primary Care / General Practice Clinic | Cohort Study | Heart failure patients | 60 | Serious |
| Fiks et al 2009^13^ | USA | Primary Care / General Practice Clinic | RCT | Children with asthma (5-19 years) | 5 | Low |
| Fiks et al 2015^14^ | USA | Primary Care / General Practice Clinic | RCT | Children with asthma (6-12 years) | 6 | Some concerns |
| Goud et al 2009^15^ | Netherlands | Hospital | RCT | Patients requiring cardiac rehabilitation due to heart surgery, acute coronary syndrome, angina or percutaneous coronary intervention, and other heart disease. |  | Some concerns |
| Kattan et al 2006^16^ | USA | Primary Care / General Practice Clinic, Hospital, Community | RCT | Children with moderate-severe asthma (5-11 years) | 12 | Low |
| Kline et al 2014^17,18^ | USA | Hospital | RCT | Adults with chest pain and dyspnea, nondiagnostic ECGs, and no obvious diagnosis. | 3 | Low |
| Kraai et al 2016 ^19^ | Netherlands | Hospital | RCT | Heart failure patients | 9 | Low |
| Kuhn et al (Adults) 2015^20^ | USA | Hospital | Case Control | Children asthma patients | 12 | Low |
| Kuhn et al (Children) 2015^20^ | USA | Hospital | Case Control | Children asthma patients | 12 | Low |
| Kuilboer et al 2006^21^ | Netherlands | Primary Care / General Practice Clinic | RCT | Asthma and COPD patients | 10 | Low |
| Martens et al 2007^22^ | Netherlands | Primary Care / General Practice Clinic | RCT | Asthma, COPD, dyslipidemia, antibiotic prescribed patients | 12 | Some concerns |
| McCowan et al 2001^23^ | United Kingdom | Primary Care / General Practice Clinic | RCT | Asthma patients (30 randomly selected from each GP site) | 6 | High |
| McKie et al 2019^24^ | USA | Primary Care / General Practice Clinic | RCT | Heart failure with reduced ejection fraction | 7 | Some concerns |
| Morganroth et al 2016^25^ | USA | Primary Care / General Practice Clinic | Pre-Post study | COPD patients | 12 | Serious |
| Poels et al 2009^26^ | Netherlands | Primary Care / General Practice Clinic | RCT | Chronic respiratory disease patients |  | Some concerns |
| Porter et al 2006^27^ | USA | Hospital | Pre-Post study | Children (1-12 years) with asthma or chronic wheezing | 5 | Some concerns |
| Rahimi et al 2020^28^ | United Kingdom | Primary Care / General Practice Clinic | RCT | Heart failure patients |  | Low |
| Rasmussen et al 2005 (versus GP usual care)^29^ | Denmark | Primary Care / General Practice Clinic | RCT | Asthma patients | 6 | Low |
| Rasmussen et al 2005 (versus Specialist usual care)^29^ | Denmark | Primary Care / General Practice Clinic, Hospital | RCT | Asthma patients | 6 | Low |
| Renzi et al 2006^30^ | Canada | Primary Care / General Practice Clinic | RCT | Asthma patients | 6 | Low |
| Seol et al 2021^31^ | USA | Primary Care / General Practice Clinic | RCT | Children with asthma | 12 | Low |
| Shiffman et al 2000^32^ | USA | Hospital | Pre-Post study | Children with asthma | 0.5 | Some concerns |
| Slok et al 2016^33^ | Netherlands | Primary Care / General Practice Clinic, Hospital | RCT | COPD patients | 18 | Low |
| Subramanian et al 2004^34^ | USA | Hospital | RCT | Heart failure patients | 12 | Some concerns |
| Tamblyn et al 2015^35^ | Canada | Primary Care / General Practice Clinic | RCT | Asthma patients | 33 | Low |
| Tierney et al 2003^36^ | USA | Primary Care / General Practice Clinic | RCT | Heart failure or ischemic heart disease patients |  | Low |
| Tierney et al 2005^37^ | USA | Primary Care / General Practice Clinic | RCT | Asthma and COPD patients | 12 | Low |
| Trinkley et al 2021^38^ | USA | Primary Care / General Practice Clinic | RCT | Heart failure patients | 6 | Low |

^#^Details available in Supplementary Appendix 2.

Supplementary Table 3 – Summary of results of included studies

| Study | Intervention | Comparator | Summary of Result |
| --- | --- | --- | --- |
| **Multi-disease CDSS** | | | |
| Eccles et al 2002^11^ | CDSS for asthma and angina | Usual care | No significant differences reported on the provision of lung function tests, asthma action plans, inhaler education, beta-blockers or smoking cessation advice between the intervention and control group.  The investigators attributed a low level of software use (median active interactions with the CDSS = 0) as a likely reason for this lack of effect. |
| Kline et al 2014^17,18^ | Web-based decision support which computed pretest probability estimates for both acute coronary syndrome and pulmonary embolism and suggested clinical actions | Usual care | Statistically significant reduction in proportion of those exposed to >5 mSv radiation to the chest in patients with no significant cardiopulmonary diagnosis within 90 days (33% vs 25%, p=0.038).  No significant difference in median length of stay in the ED was reported, median length of stay in the hospital was significantly lower in the intervention group (7.7 hours [IQR 4.0–27.3] vs 8.9 hours [IQR 4.8–29.6], p=0.046). |
| Martens et al 2007^22^ | Reactive computer reminder system (CRS) providing reminders about antibiotics, asthma/COPD prescriptions and cholesterol prescriptions | Usual care | Large variations between GP’s prescribing behaviour which underpowered the study.  No significant differences between groups were found for indicators relating to recommendations to prescribe certain drugs.  Prescribing changed and there was a fall in prescriptions of inhaled corticosteroids for newly diagnosed COPD patients older than 40 years in the intervention compared to control group (0.0 [95% CI: 0.0–0.1] vs 0.5 [95% CI:0.3–0.9], p<0.01). |
| McKie et al 2019^24^ | Intra-EHR advisory CDSS. The CDSS determined if they had heart failure with reduced ejection fraction, hyperlipidemia, or atrial fibrillation based on natural-language processing of the problem list matched with conditions in ICD-9; and if so, was the patient receiving guideline-recommended treatment. | Usual care | Higher proportion of heart failure patient visits in which treatment discrepancy was resolved (12% vs 1.9%, OR=7.57 [95% CI 1.21-47.49], p=0.03) in the intervention versus usual care groups. |
| Poels et al 2009^26^ | Computerised spirometry expert interpretation support group | Usual care | No significant differences in the proportions of changed diagnosis between both groups (OR 0.72, 95% CI 0.45 - 1.15).  No significant differences were reported for secondary outcomes - referral rate; ordering of additional diagnostic tests; changes in respiratory medication (i.e., use of short-acting bronchodilators and/or long-acting bronchodilators and/or inhaled steroids). |
| Tierney et al 2003^36^ | Computer-based cardiac care suggestions based on suggestions from local cardiologists and general internists | Usual care | No effect of CDSS on physicians’ adherence to the care recommendations, and no significant differences in quality of life, medication compliance, health care utilization, or satisfaction between groups. |
| Tierney et al 2005^37^ | Computer-generated paper encounter form that included a list of active medications for asthma and COPD | Usual care | No significant differences between groups in adherence to care suggestions.  No significant difference in quality of life and medication compliance as well as number of emergency department visits and hospitalizations between groups. |
| **Asthma CDSS (Children)** | | | |
| Bell et al 2010^3^ | Decision-support alerts and reminders to use asthma management tools  Asthma management tools available include - pediatric asthma-control tool (PACT), standardised documentation, order sets and patient asthma plan. | Passive asthma management decision support | Significant increase in controller medication prescription in the CDSS compared to usual care control group (6.7% vs 1.1%).  Significantly higher increase was also found on adherence with spirometry recommendations in the CDSS versus control group (8% vs 3.1%).  The proportion of children with an up-to-date asthma care plan significantly improved in the suburban sites but not urban sites. |
| Carroll et al 2012^5^ | CHICA asthma module - parents were asked about asthma symptoms on a prescreener form. If a parent answered yes, then the physician received a prompt to determine whether an asthma diagnosis was appropriate. | Usual care - parents received no screening questions, and physicians received no prompt. | Statistically significant 48% relative increase (2.8% net increase) in proportion of patients diagnosed with asthma in the CDSS arm compared to control group (p<0.02). |
| Choi et al 2007^8^ | Computer program that presents a total symptom score based on 11 questions provided to patients. | Spirometry and methacholine bronchial provocation test (MBPT) | Area under the curve of 0.647±0.033 for diagnosing asthma.  At the investigator identified optimum cut off total symptom score of ≥4, sensitivity was 85.2% but specificity was only 25% in ascertaining a diagnosis of asthma. |
| Dexheimer et al 2014^10^ | Patient eligibility identification by a probabilistic disease detection system (Bayesian network) combined with an asthma management system | Usual care | No significant difference in admission, triage time, length of stay and take-home asthma prescription between the CDSS and usual care control group. |
| Fiks et al 2009^13^ | Electronic health record-based clinical alerts for influenza vaccine | Usual care | No significant difference in the CDSS compared to usual care group in influenza vaccination |
| Fiks et al 2015^14^ | MyAsthma, an EHR-linked patient portal supporting shared decision-making for pediatric asthma | Usual care | Improvement in patient reported outcomes such as missing days from work and quality of life  Pilot study and no statistical testing was done between groups. |
| Kattan et al 2006^16^ | Bimonthly computer-generated letters on patient reported specific symptoms, medication, and health service use combined with guideline-based recommendations (to step up or step down) | Usual care | No difference in maximum symptom days over the past 2 weeks between the intervention and usual care control group (p=0.54). |
| Kuilboer et al 2006^21^ | Intra-EHR AsthmaCritic CDSS - provides patient specific feedback based only on data from the electronic patient record | Usual care | Statistically significant increase in the number of peak-flow measurements per patient per practice (p=0.016) and average FEV-1 measurements per patient (p=0.028) compared to the usual care control group in children <12 years old. |
| Kuhn et al (Children) 2015^20^ | Intra-EHR electronic asthma action plan decision support tool (eAAP) | Propensity score matched controls | Significantly reduced asthma exacerbations and the odds of requiring outpatient oral steroid during the 12 months follow up by 33% (p<0.001) compared with a historical propensity score matched control arm, although there were no significant differences in ED visits and hospitalisation rates. |
| Porter et al 2006^27^ | Patient-driven decision-support technology (asthma kiosk) which produces tailored plans of action for use by clinical providers | Usual care | No significant difference in provider prescription behaviour before and after CDSS implementation |
| Seol et al 2021^31^ | Artificial Intelligence based CDSS - Asthma-Guidance and Prediction System (A-GPS) | Usual care | No difference in acute exacerbation (AE) frequency between the two groups (12% for the intervention group vs. 15% for the control group, Odds Ratio: 0.82; 95%CI 0.374–1.96; p = 0.626).  Timelier follow-up after an AE by the clinical care team in the CDSS compared the control group, this difference was not statistically significant (HR = 1.93; 95% CI: 0.82–1.45, p = 0.10).  No difference between groups in the duration of well-controlled asthma during the study period. |
| Shiffman et al 2000^32^ | Handheld, computer-based decision support device | Usual care | No significant difference in provider prescription behaviour before and after CDSS implementation  Significantly higher proportion in the CDSS intervention phase receiving oxygen saturation measurement compared to during the control phase (56% vs 29%, p=0.007).  No significant differences in ED visits or hospitalisation were however found between both phases. |
| Asthma CDSS (Adults) | | | |
| Ahmed et al 2016^1^ | My Asthma Portal - tailored education, personal health record, tracking, behavior change support and link to nurse case manager | Usual care - ongoing asthma care from their pulmonologist throughout the trial, and an asthma nurse provided education and follow-up sessions as needed | Greater improvements in Mini-Asthma Quality of Life Questionnaire (MAQLQ) in the intervention compared to usual care control group, however this was statistically not significant.  No difference was found on the proportion with poor control status between groups.  Higher proportion of individuals in the intervention group had an improvement in the MAQLQ, ACT, EQ-VAS, PHQ-9 and Self-Efficacy score at 6 and 9 months compared to the control group. Only the difference in depression (PHQ) at 6 months was statistically significant. |
| Cherney et al 2021^6^ | Alert CDSS indicating the frequency of “ED/Urgent Care visit(s) within 1year, with the use of ED asthma protocol order set.” for elligible patients then recommending guideline based therapy. | Pre-intervention | Adherence to ICS prescription guidelines remained high when compared with the control phase prior to CDSS implementation (74% vs 0%, p<0.0001). No significant differences in adherence to ICS prescription were reported between the period with and without financial incentive. |
| Cho et al 2010^7^ | Computer-based asthma management program (Easy Asthma Management program, EAM program) |  | Compared to the control period before CDSS intervention, the fraction of prescriptions for oral β2-agonists, oral methylxanthines, and systemic steroids (oral or injection forms) significantly decreased after the EAM study (p=0.02, p<0.001, p <0.001, respectively).  Proportion receiving ICS prescription increased by 86%.  Significant improvements in mean self-assessed asthma symptom score from −0.54 in the first follow-up visit to 0.06 in the third follow-up visit (p<0.001) in patients followed by GPs. |
| Kuilboer et al 2006^21^ | Intra-EHR AsthmaCritic CDSS - provides patient specific feedback based only on data from the electronic patient record | Usual care | Significantly increased the average number of peak-flow measurements per patient per practice and led to a decrease in the average number of cromoglycate prescriptions in accordance with guidelines in the AsthmaCritic group (paired Wilcoxon signed-rank test, p=0.033) for the 12-39 years age group. |
| Kuhn et al (Adults) 2015^20^ | Intra-EHR electronic asthma action plan decision support tool (eAAP) | Propensity score matched controls | No significant difference in exacerbation outpatient oral steroid use, ED visit and hospitalisation in the prior 12 months. |
| McCowan et al 2001^23^ | Asthma Crystal Byte - provide decision support relating to current asthma guidelines and based on the data entered at consultation by the user | Usual care | Significant reduction in number of exacerbations (OR=0.43, 95% CI 0.21– 0.85) and GP consultations (OR=0.59, 95% CI 0.37–0.95) 6 months post intervention but not hospitalisation. |
| Rasmussen et al 2005 (versus GP usual care)^29^ | Physician-managed online interactive asthma monitoring tool | Usual care | CDSS arm improved symptoms, AQLQ score, FEV-1 value and airway hyperresponsiveness. ICS prescription, compliance with guidelines and use of asthma action plan also significantly improved in the CDSS group |
| Rasmussen et al 2005 (versus Specialist usual care)^29^ | Physician-managed online interactive asthma monitoring tool | Usual care | Similar results to GP arm except for airway hyperresponsiveness wherein the improvement was not significantly different between groups. |
| Renzi et al 2006^30^ | Memory aid - self-inking paper stamp checklist summarizing CPG criteria and guidelines for assessing asthmatic patient control and therapy. | Usual care | Significantly reduced the proportion of ED visits (9.4% vs 13.5%) compared to the usual care control group. Hospitalisations were also significantly lower in the intervention group (2.4% vs 4%). |
| Tamblyn et al 2015^35^ | Intra-EHR asthma decision support system - CDSS triggered automatically based on specific clinical codes and generate patient specific recommendations. Prepopulates each patient’s file with information on demographics, drugs, health problems, and medical visits from provincial health insurance databases | Usual care | Significant increase in the ratio of doses of inhaled corticosteroid use to fast-acting beta-agonist (0.93 vs. 0.69: difference: 0.27; 95% CI: 0.02–0.51; p=0.03) in the intervention group.  No statistically significant difference between overall uncontrolled asthma rate compared to the control group (rate difference= -8.7, 95% CI -24.7 to 7.3, p=0.29)  Reduced event rate (composite of ER visit, hospitalization, diagnosis with a closely related respiratory condition, and excessive use of fast-acting bronchodilators) of 28.4 per 100 person years (95% CI -55.6 to -1.2, p=0.04) in those with baseline poor control. |
| **COPD CDSS** | | | |
| Morganroth et al 2016^25^ | CareManager - case management, academic detailing, and decision support assistance | Usual care | No significant difference was found for ED exacerbations.  Total exacerbations, inpatient exacerbations, and outpatient exacerbations were all significantly lower post intervention. |
| Slok et al 2016^33^ | Assessment of Burden of COPD (ABC) tool - short validated questionnaire assessing the experienced burden of COPD, objective COPD parameter (eg, lung function) and a treatment algorithm including a visual display and treatment advice | Usual care | Higher proportion of patients with clinically relevant improvement for the St. George’s Respiratory Questionnaire (SGRQ) in the intervention compared to control group (OR 1.85, 95% CI 1.08 to 3.16).  No significant differences in COPD Assessment Test (CAT) score was found between groups. |
| **Heart Failure CDSS** | | | |
| Breathett et al 2018^4^ | Nurse practitioner (NP) education plus tablet application (interactive conditional logic program that flags patient questions to medical staff) | NP education only | Lower but statistically non-significant all-cause 30-day readmission for the CDSS group compared to the control group. |
| Choi et al 2020^9^ | Artificial Intelligence based CDSS | Usual care | Significantly higher accuracy of the CDSS compared to usual care (97.9% vs 76.3%).  The usual care group had an equal accuracy with the CDSS on ruling out heart failure, however the CDSS group had better accuracy in diagnosis HFrEF (100% vs 66.6%), HFmrEF (100% vs 33%) and HFpEF (100% vs 55%) compared to the usual care group. |
| Esposti et al 2012^12^ | Intra-EHR automatic reminder to help general practitioners (GPs) to identify HF patients and to prescribe them with recommended drugs. | Usual care | Proportion of patients with greater than or equal to 2 hospitalisations between the intervention and control group to be no different (33.3% vs 33.1%) between the groups.  They noted that providers agree to less than half of care suggestions. |
| Goud et al 2009^15^ | Intra-EHR CARDSS (cardiac rehabilitation decision support system) - conduct assessment of patients with structured dialogues and formulate patient specific rehabilitation program. On request, users can see the rationale behind recommendations and links to research evidence | Usual care with passive access to guidelines | Intervention group had a higher concordance with guideline recommendation for exercise (92.6% vs 84.7%), education (87.6% vs 63.9%), relaxation (59.6% vs 34.1%) and lifestyle change (57.4% vs 54.1%) compared to control.  For all guideline recommendations, the intervention arm showed lesser undertreatment (withholding treatment from patients who should receive it) and lesser overtreatment (treatment of patients who should be left untreated) compared to control arm. |
| Kraai et al 2016 ^19^ | Information and Computing Technology-guided-disease-management-system (ICT-guided-DMS) | ICT-guided-DMS with telemonitoring | No statistically significant difference in the CDSS only group compared to CDSS with telemonitoring for the mean primary composite endpoint, all cause outpatient clinic visit, hospitalisation, heart failure readmission and all-cause mortality.  Health related QoL improved in both groups and did not significantly differ but the telemonitoring group had a significantly lower number of heart failure related outpatient clinic visits (median 2 vs 4, P=0.02). |
| Subramanian et al 2004^34^ | Intra-EHR care suggestions generated with electronic medical record data and symptom data obtained from questionnaires mailed to patients within 2 weeks of scheduled outpatient visits | Intra-EHR care suggestions generated with electronic medical record data alone | Higher all-cause hospitalizations at 6 months (1.5±1.1 vs. 0.7±0.4 hospitalizations, p=0.0002) and 12 months (2.3±1.2 vs. 1.7±0.7 hospitalizations, p=0.05).  No significant changes in NYHA class (p=0.1) and SF-36 quality-of-life measures (p=0.1), as well as no differences in the number of outpatient visits (GP, cardiologists, and ED visit) between intervention and control patients (6.7 vs. 7.1 visits, p=0.48) were reported.  No significant differences in adherence to care suggestions between physicians in the intervention and control groups (33% vs. 30%, p=0.4), potentially explaining these paradoxical outcomes. |
| Rahimi et al 2020^28^ | CDSS for GPs with patient home telemonitoring | Home telemonitoring | Addition of centralised support did not significantly increase the proportion receiving guideline-recommended medical therapy, mean physical well-being of participants, New York Heart Association (NYHA) score, MAGGIC score, brain natriuretic peptide (BNP) and NT-pro-BNP levels.  No significant difference for death, hospital admissions, cardiovascular death and cardiovascular hospital admissions were found. |
| Trinkley et al 2021^38^ | Enhanced CDSS alerts informed by PRISM IS Framework | Commercially available CDSS alerts | Adoption and effectiveness of the enhanced alert were significantly higher than those of the commercial alert (62% vs 29% alerts adopted, p<0.001; 14% vs 0% changed prescribing, p=0.006).  Among the 21 clinicians interviewed, most stated they preferred the enhanced alert. |

Supplementary Table 4 – Summary of health economics related outcomes

| Study | Disease | Summary of Result |
| --- | --- | --- |
| Fiks et al 2015^14^ | Asthma | lower proportion of specialist visits in the intervention compared to control group (31% vs 44%) and a lower proportion of primary care asthma visits (62% vs 67%).  Greater reduction in the days of school missed in the intervention group compared to control group (-25% vs -16%) and a 42% reduction in days of work missed by parents in the intervention group against a 5% increase in days of work missed in the control group.  The authors however did not cost these potential savings. |
| Kattan et al 2006^16^ | Asthma | Lower number of school days missed and days of limited activities due to asthma in the intervention group, however these were statistically non-significant.  Statistically significant reduction in number of ED visits in the intervention compared to control group (0.87±0.07 vs 1.14±0.08, p=0.013) despite the number of unscheduled clinic visits and hospitalizations being not statistically different between both groups (P=0.014 and 0.56 respectively).  Cost benefit analysis conducted found that by year 1 the savings from the reduction in ED visits was sufficient to provide a cost-benefit. Montecarlo simulation suggests a 97% chance of this cost-saving. The intervention was estimated to cost USD69.20 per child over the year. When this cost was added to the cost of health services use for the year by intervention children and compared with the cost of health service use by control children, there was a savings of USD337 per child in the intervention group. |
| Kline et al 2014^17,18^ | Breathlessness | Significantly lower median costs for medical care within 30 days in the intervention compared to control group (USD2761± 7965 vs USD 3933±12639 respectively, p=0.027).  Significantly lower median total charge of medical care within 30 days reported (USD 11916±26288 vs USD 16413±38993, p=0.008) as well.  The investigators also conducted a modelling study^17,18^ to evaluate the lifetime cost benefit of the CDSS intervention. Markov microsimulation for a 40-year-old patient receiving the intervention found lifetime cost savings of USD 497 for women and USD 528 for men, associated with small gains in QALYs (2 and 6 days, respectively) and lower rates of cancer mortality in both sexes, but a reduction in ICH only in males (not cancer, pulmonary embolism [PE], acute coronary syndrome [ACS], renal failure and ischemic stroke). Those aged 60 years was predicted to continue to save costs and reduce mortality from both ICH and cancer. |
| Kraai et al 2016 ^19^ | Heart disease | Mean total costs was EUR5006 (USD5650 as of Jan 2022) per patient for the ICT-guided-DMS group and EUR6366 (USD7185 as of Jan 2022) per patient for the telemonitoring group (EUR1360 [USD1535 as of Jan 2022] in favour of ICT-guided-DMS group). |
| Kuilboer et al 2006^21^ | Asthma | 5-10% increase in number of contacts with providers in the intervention compared to control group as well as increase in number of pulmonary function tests with providers in the intervention compared to control group. |
| McCowan et al 2001^23^ | Asthma | Proportion of patients in the intervention group who initiated an asthma consultation with their general practice was lower than that in the control group (22% vs 34%, OR=0.59, CI 0.37–0.95).  No evidence of a difference in the proportion of patients attending practice-initiated reviews for their asthma (33% vs 42%, OR=0.69, CI 0.21-2.21). |
| Seol et al 2021^31^ | Asthma | Mean health care costs of children during the trial (compared to before the trial) in the intervention group were lower than those in the control group (-USD1,036 95% CI -USD2177 to USD44 for the intervention group vs. +USD80 95% CI -USD841 to USD1000 for the control group), though this was not statistically significant (P = 0.12). |
| Shiffman et al 2000^32^ | Asthma | Higher costs in the CDSS than control group (USD145.61 vs USD103.11). |
| Tierney et al 2003 ^36^ | Heart disease | Total health charges were lower for the intervention compared to control group (USD6302±10928 vs USD7025±17024 respectively), this was not statistically significant due to the wide variation in outpatient and inpatient costs reported in the study. |
| Tierney et al 2005^37^ | Asthma | Intervention group had a significantly higher total healthcare costs (USD8,006±18,720 vs USD5,800±8,536) mainly contributed by the substantially higher inpatient costs in the intervention compared to control group (USD4,864±17,257 vs USD2,671±6,805 respectively). |

Supplementary Table 5 – Summary of impact on providers

| Study | Disease | Summary of Result |
| --- | --- | --- |
| Cherney et al 2021^6^ | Asthma | Found that once financial incentives were rolled back, use metrics remain not significantly different throughout the 12 months follow up |
| Cho et al 2010^7^ | Asthma | Proportion of physicians who responded “yes” to the question “Should inhaled corticosteroid be prescribed to reduce airway inflammation?” significantly increased from 83.3% before to 95.8% after participating in their CDSS study (p=0.005).  84.3% of participants agreed that the CDSS program was easy to follow and thus more practical than currently used written guidelines, and 88.5% would recommend the CDSS program to their colleagues. |
| Choi et al^9^ | Asthma | Significantly reduced time for reviewing EHRs for asthma management of each participant (median: 3.5 min, IQR: 2–5), compared to usual care without CDSS (median: 11.3 min, IQR: 6.3–15); p<0.001). |
| Eccles et al 2002^11^ | Asthma and Angina | Low levels of CDSS use and concluded that it was unclear whether there are benefits from integrating such systems into clinical encounters where busy practitioners manage patients with complex and multiple conditions |
| Esposti et al 2012^12^ | Heart failure | Providers agree with less than half of computer-generated care suggestions from evidence-based HF guidelines most often because the suggestions were felt to be inapplicable to their patients or unlikely to be tolerated |
| Kuilboer et al 2006^21^ | Asthma | Noted that in the CDSS arm there were changes in physicians data recording habits with an increase in the ratio of coded measures to free-text entry, as the CDSS requires structured data to provide recommendations. |
| Martens et al 2007^22^ | Multi-disease | High inter-doctor variation in prescribing behaviour was found and providers were reported to be more compliant to reminders barring prescription of certain drugs rather than those that recommend certain medication. |
| McCowan et al 2001^23^ | Asthma | Visits lasted longer in the CDSS group however CDSS can still be used in consultations lasting less than 10 minutes.  management recommendations and reminders were popular with the users and were deemed to provide relevant clinical advice.  Users felt the software apply management guidelines for asthma and contributed to an improvement in the quality of the consultation. Printed management plans were of use and seemed to be of value to the patients. |
| McKie et al 2019^24^ | Heart failure | No statistically significant change in providers self-reporting being able to efficiently manage cardiac medications, the study reported a statistically significant increase in providers use of a risk estimate tool when making clinical decisions for heart failure (p<0.01).  Statistically significant increase in providers impression that CDSS save them time post intervention (p=0.01) and that CDSS allowed them to accomplish more work than would otherwise be possible (p<0.01).  CDSS not meeting patient needs  CDSS advice might be differentially needed and hypothesised more complex conditions may be more amenable to CDSS  CDSS improved used of heart failure tools, it had no significant impact on atrial fibrillation or hyperlipidemia with only 19% of CDSS alerts being reviewed. |
| Renzi et al 2006^30^ | Asthma | Improve provider knowledge score regarding asthma control criteria (4.5/8 vs 3/8), education and therapy (3.3/8 vs 2.1/8) compared to control group  74% of the physicians reported keeping the decision tool (a stamp) on their desk, 13% in a drawer and the others in different places.  84% of the physicians thought the stamp was useful in daily practice and, on average, physicians reported using the tool for 54% of their patients with asthma.  87% of physicians reported that they would continue to use the tool at the end of the study. |
| Shiffman et al 2000^32^ | Asthma | Significantly higher proportion of 30 to 60 minutes visit in the CDSS compared to control group (67% vs 46%, p=0.0004) |
| Subramanian et al 2004^34^ | Heart failure | Two-thirds of suggestions being ignored by providers despite baseline reports showing a substantial number of veterans with heart failure were not receiving care in accordance to accepted local and national guidelines.  Pre-study questionnaires showed physicians have ambivalent feelings about guidelines in general which might affect their willingness to implement recommendations. |
| Tamblyn et al 2015^35^ | Asthma | Physicians were reported to differentially use CDSS and more likely to use it in out-of-control patients |
| Tierney et al 2003^36^ | Heart disease | Physicians viewed guidelines as providing helpful information but constraining their practice and not helpful in making decisions for individual patients  Physicians may have rebelled at the notion of the computer telling them how to manage their patients |

Supplementary Table 6 – Summary of unintended consequences of CDSS use

| Study | Disease | Summary of Result |
| --- | --- | --- |
| Ahmed et al 2016^1^ | Asthma | Lowering engagement with the patient facing side of a CDSS from 100% having at least one login in week 1 to 56% in week 19. |
| Ansari et al 2003^2^ | Heart failure | Increased mean length of time from initiation to target dose compared to the control group (9.3 months vs 8.5 months) |
| Bell et al 2010^3^ | Asthma | Urban sites already having a high rate of asthma plans did not have much improvement whereas substantial improvements in suburb sites with the CDSS. |
| Goud et al 2009^15^ | Heart disease | CDSS alone is insufficient to improve concordance with guidelines when this requires additional resources—for example, in increasing uptake of lifestyle change therapy. |
| Kline et al 2014^17,18^ | Multidisease | While CDSS use reduced the rate of ACS diagnosis and death from ACS at age 40, its use was associated with an increased death rate from ACS at age 60 for both sexes. |
| Kuilboer et al 2006^21^ | Asthma | Differential effects based on age group  CDSS impacts limited by system wide constraints |
| Rasmussen et al 2005^29^ | Asthma | More unscheduled visits, a higher consumption of inhaled steroids, and more side effects. Even so, the investigators noted that CDSS use resulted in closer monitoring, immediate feedback, adequate medication, and better compliance and that these produced better asthma control in patients as reported in the study. |
| Renzi et al 2006^30^ | Asthma | Limitations of decision support tools impact in patients that are new, walk in patients or those who do not come regularly to follow up visits. |

Appendix 2. Study Risk of Bias Assessment

**Observational Studies (by RoB Vis Tool)^[[1]](#footnote-1)^**

**
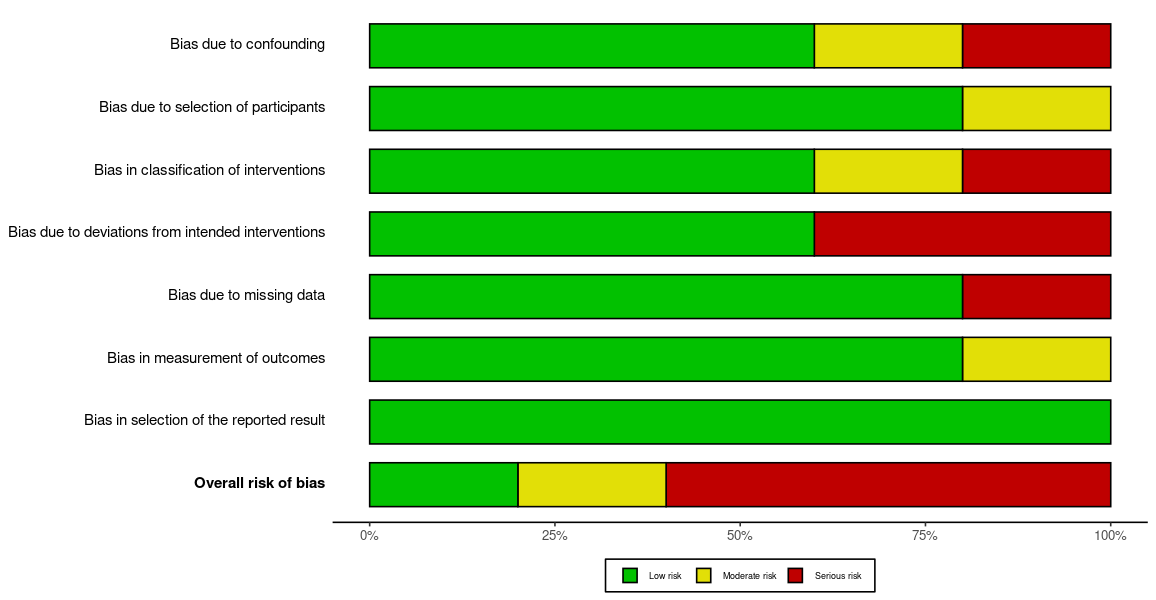
**


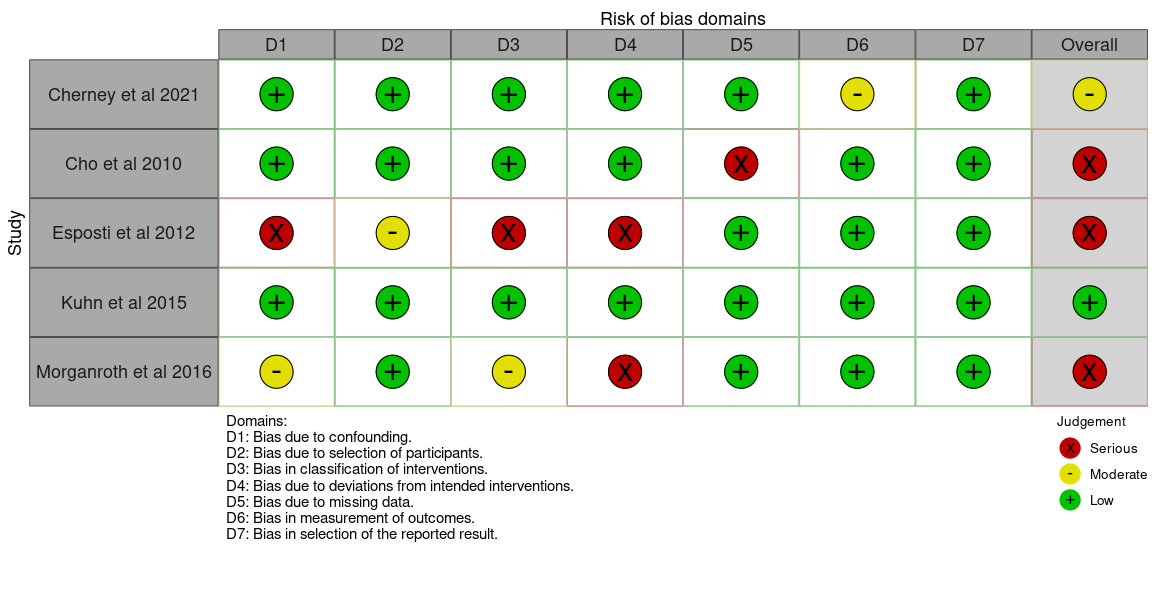


**Diagnostic Studies (by RoB Vis Tool)^[[2]](#footnote-2)^**


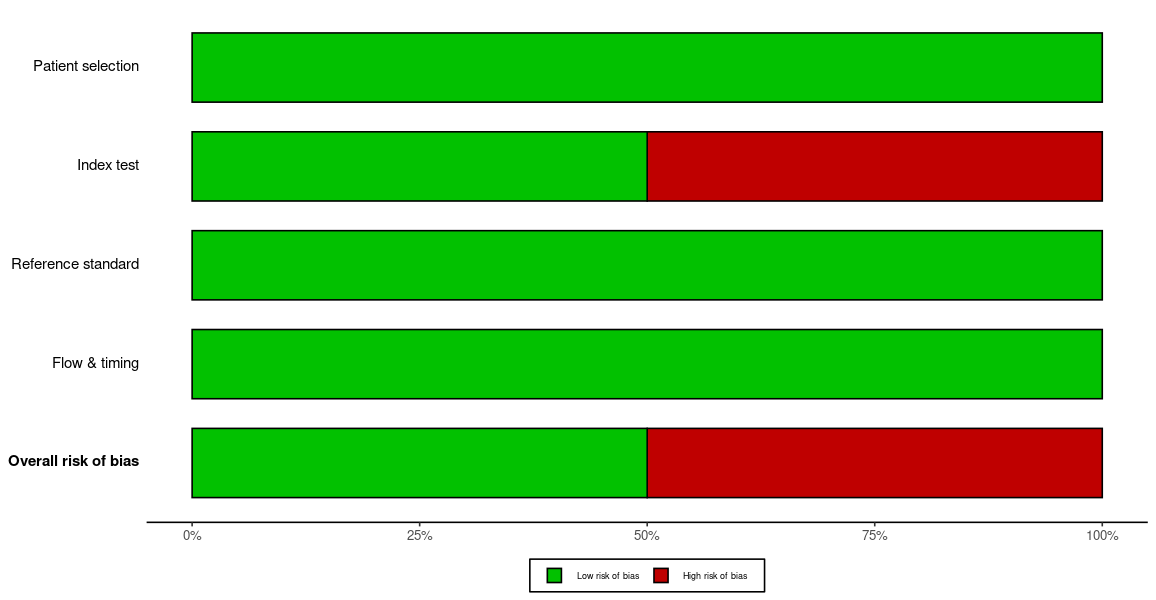

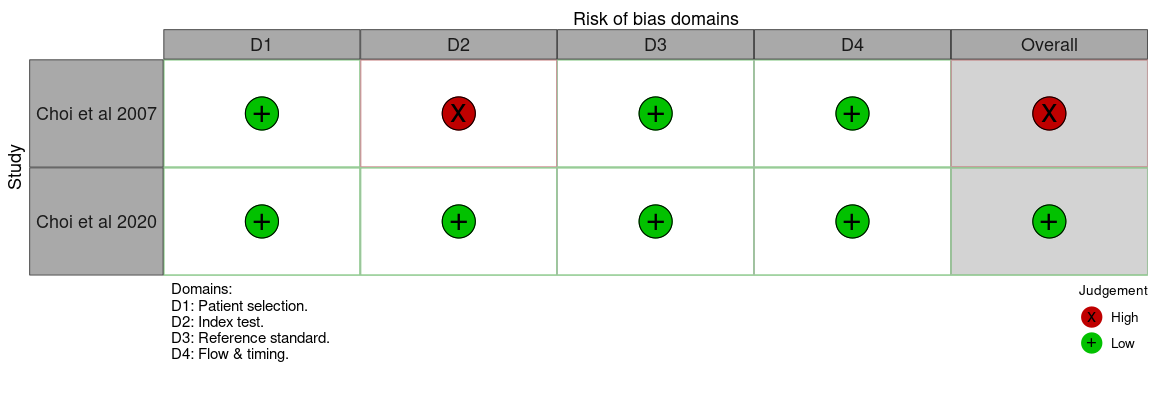


References

1 Ahmed, S. *et al.* The Effectiveness of Web-Based Asthma Self-Management System, My Asthma Portal (MAP): A Pilot Randomized Controlled Trial. *J Med Internet Res* **18**, e313, doi:10.2196/jmir.5866 (2016).

2 Ansari, M. *et al.* Improving guideline adherence: a randomized trial evaluating strategies to increase beta-blocker use in heart failure. *Circulation* **107**, 2799-2804, doi:10.1161/01.CIR.0000070952.08969.5B. Epub 2003 May 19.

3 Bell, L. M. *et al.* Electronic health record-based decision support to improve asthma care: a cluster-randomized trial. *Pediatrics* **125**, e770-777, doi:10.1542/peds.2009-1385 (2010).

4 Breathett, K. *et al.* Pilot Randomized Controlled Trial to Reduce Readmission for Heart Failure Using Novel Tablet and Nurse Practitioner Education. *Am J Med* **131**, 974-978, doi:10.1016/j.amjmed.2018.02.017. Epub 2018 Mar 16.

5 Carroll, A. E. *et al.* Increased Physician Diagnosis of Asthma with the Child Health Improvement through Computer Automation Decision Support System. *Pediatric Allergy, Immunology, and Pulmonology* **25**, 168-171 (2012).

6 Cherney, K., Bulloch, B., Mecham, C., Drewek, R. & Mirea, L. Inhaled corticosteroid prescriptions in the ED for recurrent asthma using IT clinical decision support: revisit after cessation of an incentive program. *Journal of Asthma* (2021).

7 Cho, S. H. *et al.* Effectiveness of a computer-assisted asthma management program on physician adherence to guidelines. *J Asthma* **47**, 680-686, doi:10.3109/02770903.2010.481342 (2010).

8 Choi, B. W. *et al.* Easy diagnosis of asthma: computer-assisted, symptom-based diagnosis. *J Korean Med Sci* **22**, 832-838, doi:10.3346/jkms.2007.22.5.832 (2007).

9 Choi, D., Park, J., Ali, T. & Lee, S. Artificial intelligence for the diagnosis of heart failure. *NPJ DIGITAL MEDICINE* **3** (2020).

10 Dexheimer, J. W. *et al.* Implementation and evaluation of an integrated computerized asthma management system in a pediatric emergency department: a randomized clinical trial. *Int J Med Inform* **83**, 805-813, doi:10.1016/j.ijmedinf.2014.07.008 (2014).

11 Eccles, M. *et al.* Effect of computerised evidence based guidelines on management of asthma and angina in adults in primary care: cluster randomised controlled trial. *BMJ* **325**, 941, doi:10.1136/bmj.325.7370.941 (2002).

12 Esposti, L. D. *et al.* Availability of computerised reminders in primary care doesn't reduce heart-failure repeated hospitalisations. *British Journal of Medical Practitioners* **5** (2012).

13 Fiks, A. G. *et al.* Impact of electronic health record-based alerts on influenza vaccination for children with asthma. *Pediatrics* **124**, 159-169, doi:10.1542/peds.2008-2823 (2009).

14 Fiks, A. G. *et al.* Parent-reported outcomes of a shared decision-making portal in asthma: a practice-based RCT. *Pediatrics* **135**, e965-973, doi:10.1542/peds.2014-3167 (2015).

15 Goud, R. *et al.* Effect of guideline based computerised decision support on decision making of multidisciplinary teams: cluster randomised trial in cardiac rehabilitation. *BMJ* **338**, b1440, doi:10.1136/bmj.b1440 (2009).

16 Kattan, M. *et al.* A randomized clinical trial of clinician feedback to improve quality of care for inner-city children with asthma. *Pediatrics* **117**, e1095-1103, doi:10.1542/peds.2005-2160 (2006).

17 Kline, J. A. *et al.* Multicenter, randomized trial of quantitative pretest probability to reduce unnecessary medical radiation exposure in emergency department patients with chest pain and dyspnea. *Circ Cardiovasc Imaging* **7**, 66-73, doi:10.1161/CIRCIMAGING.113.001080 (2014).

18 Troyer, J. L. *et al.* Cost-effectiveness of quantitative pretest probability intended to reduce unnecessary medical radiation exposure in emergency department patients with chest pain and dyspnea. *Acad Emerg Med* **22**, 525-535, doi:10.1111/acem.12648 (2015).

19 Kraai, I. *et al.* The value of telemonitoring and ICT-guided disease management in heart failure: Results from the IN TOUCH study. *Int J Med Inform* **85**, 53-60, doi:10.1016/j.ijmedinf.2015.10.001 (2016).

20 Kuhn, L. *et al.* Planning for Action: The Impact of an Asthma Action Plan Decision Support Tool Integrated into an Electronic Health Record (EHR) at a Large Health Care System. *J Am Board Fam Med* **28**, 382-393, doi:10.3122/jabfm.2015.03.140248.

21 Kuilboer M *et al*. Computed critiquing integrated into daily clinical practice affects physicians' behavior--a randomized clinical trial with AsthmaCritic. *Methods of information in medicine* **45**, 447 (2006).

22 Martens, J. D. *et al.* The effect of computer reminders on GPs' prescribing behaviour: a cluster-randomised trial. *Int J Med Inform* **76 Suppl 3**, S403-416, doi:10.1016/j.ijmedinf.2007.04.005 (2007).

23 McCowan, C. *et al.* Lessons from a randomized controlled trial designed to evaluate computer decision support software to improve the management of asthma. *Med Inform Internet Med* **26**, 191-201, doi:10.1080/14639230110067890 (2001).

24 McKie, P. M. *et al.* Computerized Advisory Decision Support for Cardiovascular Diseases in Primary Care: A Cluster Randomized Trial. *Am J Med* **133**, 750-756 e752, doi:10.1016/j.amjmed.2019.10.039 (2020).

25 Morganroth, M., Pape, G., Rozenfeld, Y. & Heffner, J. E. Multidisciplinary COPD disease management program: impact on clinical outcomes. *Postgrad Med* **128**, 239-249, doi:10.1080/00325481.2016.1129259 (2016).

26 Poels, P. J. *et al.* Spirometry expert support in family practice: a cluster-randomised trial. *Prim Care Respir J* **18**, 189-197, doi:10.4104/pcrj.2009.00047 (2009).

27 Porter, S. C., Forbes, P., Feldman, H. A. & Goldmann, D. A. Impact of patient-centered decision support on quality of asthma care in the emergency department. *Pediatrics* **117**, e33-42, doi:10.1542/peds.2005-0906 (2006).

28 Rahimi, K. *et al.* Home monitoring with technology-supported management in chronic heart failure: A randomised trial. *Heart* **106**, 1573-1578 (2020).

29 Rasmussen, L. M., Phanareth, K., Nolte, H. & Backer, V. Internet-based monitoring of asthma: a long-term, randomized clinical study of 300 asthmatic subjects. *J Allergy Clin Immunol* **115**, 1137-1142, doi:10.1016/j.jaci.2005.03.030 (2005).

30 Renzi, P. M., Ghezzo, H., Goulet, S., Dorval, E. & Thivierge, R. L. Paper stamp checklist tool enhances asthma guidelines knowledge and implementation by primary care physicians. *Can Respir J* **13**, 193-197, doi:10.1155/2006/825281 (2006).

31 Seol, H. Y. *et al.* Artificial intelligence-assisted clinical decision support for childhood asthma management: A randomized clinical trial. *PloS one* **16**, e0255261 (2021).

32 Shiffman, R. N., Freudigman, M., Brandt, C. A., Liaw, Y. & Navedo, D. D. A guideline implementation system using handheld computers for office management of asthma: effects on adherence and patient outcomes. *Pediatrics* **105**, 767-773, doi:10.1542/peds.105.4.767.

33 Slok, A. H. *et al.* Effectiveness of the Assessment of Burden of COPD (ABC) tool on health-related quality of life in patients with COPD: a cluster randomised controlled trial in primary and hospital care. *BMJ Open* **6**, e011519, doi:10.1136/bmjopen-2016-011519 (2016).

34 Subramanian, U. *et al.* A controlled trial of including symptom data in computer-based care suggestions for managing patients with chronic heart failure. *Am J Med* **116**, 375-384, doi:10.1016/j.amjmed.2003.11.021 (2004).

35 Tamblyn, R. *et al.* Evaluating the impact of an integrated computer-based decision support with person-centered analytics for the management of asthma in primary care: a randomized controlled trial. *J Am Med Inform Assoc* **22**, 773-783, doi:10.1093/jamia/ocu009 (2015).

36 Tierney, W. M. *et al.* Effects of computerized guidelines for managing heart disease in primary care. *J Gen Intern Med* **18**, 967-976, doi:10.1111/j.1525-1497.2003.30635.x (2003).

37 Tierney, W. M. *et al.* Can Computer-Generated Evidence-Based Care Suggestions Enhance Evidence-Based Management of Asthma and Chronic Obstructive Pulmonary Disease? A Randomized, Controlled Trial. *Health services research* (2005).

38 Trinkley, K. *et al.* Applying Clinical Decision Support Design Best Practices With the Practical Robust Implementation and Sustainability Model Versus Reliance on Commercially Available Clinical Decision Support Tools: Randomized Controlled Trial. *JMIR Med Inform* **9** (2021).

1. McGuinness, LA, Higgins, JPT. Risk-of-bias VISualization (robvis): An R package and Shiny web app for visualizing risk-of-bias assessments. Res Syn Meth. 2020; 1- 7. https://doi.org/10.1002/jrsm.1411 [↑](#footnote-ref-1)
2. McGuinness, LA, Higgins, JPT. Risk-of-bias VISualization (robvis): An R package and Shiny web app for visualizing risk-of-bias assessments. Res Syn Meth. 2020; 1- 7. https://doi.org/10.1002/jrsm.1411 [↑](#footnote-ref-2)
